# Mesenchymal Stem Cell-Derived Exosomes Exhibit Promising Potential for Treating SARS-CoV-2-Infected Patients

**DOI:** 10.3390/cells10030587

**Published:** 2021-03-07

**Authors:** Alok Raghav, Zeeshan Ahmad Khan, Viabhav Kumar Upadhayay, Prashant Tripathi, Kirti Amresh Gautam, Brijesh Kumar Mishra, Jamal Ahmad, Goo-Bo Jeong

**Affiliations:** 1 Multidisciplinary Research Unit, Department of Health Research, Ministry of Health and Family Welfare, GSVM Medical College, Kanpur 208002, Uttar Pradesh, India; alokalig@gmail.com (A.R.); mru.gsvm@gmail.com (P.T.); emails2kirti@gmail.com (K.A.G.); 2 Department of Bioengineering, Korea University of Technology and Education, Cheonan-si 31253, Korea; zakkhan09@gmail.com; 3 Department of Agriculture, Invertis University, Bareilly-243123, Uttar Pradesh, India; viabhav.amu@gmail.com; 4 Department of Endocrinology, UCMS, GTB Hospital, Dilshad Garden, Delhi 110095, India; brijeshgtbh@gmail.com; 5 Rajiv Gandhi Centre for Diabetes and Endocrinology, J.N Medical College, Aligarh Muslim University, Aligarh 202002, Uttar Pradesh, India; jamalahmad11@rediffmail.com; 6 Department of Anatomy and Cell Biology, College of Medicine, Gachon University, 155 Getbeol-ro, Yeonsu-gu, Incheon 21999, Korea

**Keywords:** exosomes, SARS-CoV-2, COVID-19, mesenchymal stem cells, anti-inflammatory, extracellular vesicles

## Abstract

The novel coronavirus severe acute respiratory syndrome-CoV-2 (SARS-CoV-2) is responsible for COVID-19 infection. The COVID-19 pandemic represents one of the worst global threats in the 21st century since World War II. This pandemic has led to a worldwide economic recession and crisis due to lockdown. Biomedical researchers, pharmaceutical companies, and premier institutes throughout the world are claiming that new clinical trials are in progress. During the severe phase of this disease, mechanical ventilators are used to assist in the management of outcomes; however, their use can lead to the development of pneumonia. In this context, mesenchymal stem cell (MSC)-derived exosomes can serve as an immunomodulation treatment for COVID-19 patients. Exosomes possess anti-inflammatory, pro-angiogenic, and immunomodulatory properties that can be explored in an effort to improve the outcomes of SARS-CoV-2-infected patients. Currently, only one ongoing clinical trial (NCT04276987) is specifically exploring the use of MSC-derived exosomes as a therapy to treat SARS-CoV-2-associated pneumonia. The purpose of this review is to provide insights of using exosomes derived from mesenchymal stem cells in management of the co-morbidities associated with SARS-CoV-2-infected persons in direction of improving their health outcome. There is limited knowledge of using exosomes in SARS-CoV-2; the clinicians and researchers should exploit exosomes as therapeutic regime.

## 1. Introduction

The novel coronavirus disease-2019 was also termed COVID-19 by the World Health Organization (WHO) on 11 February 2020 [1]. The International Virus Classification Commission (IVCC) announced the novel coronavirus as Severe Acute Respiratory Syndrome Coronavirus 2 (SARS-CoV-2). Severe acute respiratory syndrome (SARS) and Middle East respiratory syndrome (MERS) were also among the deadliest viral infections that have been previously identified [2]. COVID-19 is caused by SARS-CoV-2, and this disease became a devastating global pandemic within only a few months. Currently, COVID-19 cases have been reported in numerous countries throughout the world. According to the latest World Health Organization (WHO) data up to 19 January 2021, the number of confirmed cases is 93,956,883 with confirmed deaths of 2,029,084 individuals in 216 different countries [3]. The WHO also listed COVID-19 as a Public Health Emergency of International Concern (PHEIC). The relative ease of COVID-19 transmission and the mortality, severe illness, and disruption of socioeconomic structures caused by this disease make this illness a source of major concern to the global population. The World Trade Organization (WTO) announced that there was a decline in world trade of between 13% and 32% in 2020 due to the COVID-19 pandemic [4]. Due to the large amount of public interest in the development of new and effective drugs, and therapies for the treatment of COVID-19, a number of researchers are investigating and promoting stem-cell or exosome-based interventions that may ultimately prove useful for the treatment of COVID-19 or SARS-CoV-2. However, the use of stem cell- or exosome-based therapy has not been approved by the US Food and Drug Administration (FDA). A recently published study demonstrated that MSCs mediate the inflammatory response and can be effective for the treatment of acute lung injury (ALI) and acute respiratory distress syndrome (ARDS) [5]. The literature also suggests that MSCs can be effectively used for the treatment of critical COVID-19 cases [5]. The success of this type of treatment is based on the ability of MSC-derived exosomes to elicit a therapeutic response by balancing inflammation via the paracrine signaling pathway [5]. The present review emphasizes the proposed detailed mechanism underlying the use of exosomes derived from mesenchymal stem cells in the treatment of COVID-19, and a number of insights into the mechanisms of SARS-CoV-2-related pathogenesis are also provided.

## 2. Pathological Features of COVID-19 

The primary pathological features of SARS-CoV-2 caused by the COVID-19 pandemic are hypoxemia, chronic pulmonary inflammation and edema, and diffuse alveolar damage with exudates rich in cellular fibromyxoid and hyaline membrane architecture [6]. Previous studies have reported that these pathological changes are similar to those observed in response to ALI, ARDS, severe acute respiratory syndrome (SARS), and Middle Eastern respiratory syndrome (MERS) [6]. However, SARS-CoV-2 or COVID-19 is characterized by more severe inflammatory exudation, inflammatory cytokine storm, pulmonary edema, and milder pulmonary fibrosis [6]. Due to its high transmission properties and high sustainability in the environment, the disease spreads at an immensely rapid rate. Transmission is achieved through aerosols containing mucus and though fecal medium [1,7]. This virus can survive for up to 3 h in the air if an infected person sneezes without covering their face. Once the pathogen is inoculated into the respiratory system, it binds to alveoli in a manner similar to that of SARS-CoV [8,9]. The virus exhibits a very high affinity for type 2 pneumocytes within the alveoli. The main role of these type 2 pneumocytes is to release surfactants that reduce the surface tension of the alveoli sac and decrease the collapsing pressure, which is one of the key events in respiration. COVID-19 is further classified into different clinical stages based on the cell types that are likely to be infected [10].

### 2.1. Asymptomatic (1-2 Day of Post-Infection)

**Figure 1:**
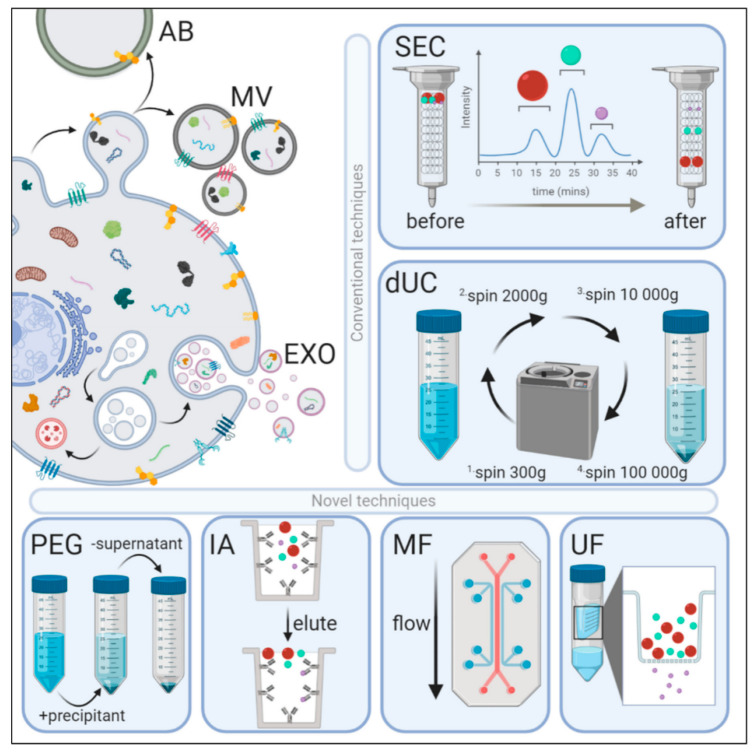
Extracellular vesicles (EVs) biogenesis and methods of isolation of exosomes. AB: Apoptotic bodies, microvesicles (MV), exosomes (EXO), size-exclusion chromatography (SEC) and differential ultracentrifugation (dUC), Poly-ethylene glycol (PEG), Microfluidics (MF) and Ultrafiltration (UF). Adapted under the terms and conditions of the Creative Commons Attribution (CC BY) license (http://creativecommons.org/licenses/by/4.0/) (accessed on 2 March 2021) [15].

SARS-CoV-2 enters the nasal passage where it binds to the epithelial cells of the nasal cavity and begins replicating. The putative entry receptors used by SARS-CoV-2 are angiotensin-converting enzyme 2 (ACE-2) receptors. The coronavirus genome encodes four major structural proteins that include the S protein for spikes, the N protein for the nucleocapsid, the M protein for the membrane, and the E protein for the envelope. The pathogen possesses positive-sense single-stranded (+SS) RNA [11], as indicated in Figure 1. The S protein is responsible for the entry and binding of SARS-CoV-2 to ACE-2 receptors in epithelial cells. Once the virus enters the host cell, it shreds its protein portion and RNA is subsequently released into the cytoplasm. SS (+) RNA uses ribosomes to translate the various polypeptides required for the formation of spike proteins, envelope proteins, and capsids. Furthermore, SS (+) RNA uses host RNA-dependent RNA polymerase to form multiple copies. It has been previously reported that ACE2 is the primary receptor for both SARS-CoV-2 and SARS-CoV [12,13]. A previously published in vitro study demonstrated that SARS-CoV infects the ciliated cells of the conducting airways [14]. Observations indicate that there is a local progression of the virus in the mild version, and thus, limited innate immunity is observed. At this stage of pathogenesis, the viral load can be screened using a nasal swab and subsequent real-time PCR (RT-PCR). During this stage of transmission, nasal swabs may be more sensitive than throat swabs. 

### 2.2. Upper Airway and Conducting Airway Response

During this stage of transmission, the virus propagates and travels down the respiratory tract through the conducting airways to thereby trigger a more robust innate immune response, and at this stage, viral particles are present in nasal swabs and sputum. This stage represents the clinical manifestations of COVID-19. The polypeptide produced by the pathogen is converted into its active form, and through the function of the endoplasmic reticulum, various components of the pathogen are assembled and then released through exocytosis processes. The virus spreads down the bronchial tubes and infects neighboring cells. The burst causes release signals that attract macrophages that further release various inflammatory markers such as IL-1, IL-6, and TNF alpha into the alveolar space. The presence of β- and ƛ-interferon confirms viral transmission in epithelial cells [16]. It has been reported that 80% of infected individuals exhibit restricted infection in the upper and conducting airways only, and these individuals can be treated at home using conservative symptomatic therapies [10].

### 2.3. Hypoxia and Progression to Acute Respiratory Distress Syndrome (ARDS)

Unfortunately, 20% of infected patients will progress to stage 3 transmission of COVID-19, where they develop pulmonary infiltrations that can result in life-threatening complications. At this stage, the SARS-CoV virus traverses through the gas exchange units of the lungs and infects alveolar type II cells in a manner similar to that of the influenza virus [17,18]. SARS-CoV propagates in type II cells, thus enabling them to undergo apoptosis [19]. It is evident that SARS and COVID-19 both cause diffuse alveolar damage in conjunction with impairment of fibrin-rich hyaline membranes and the appearance of a number of multinucleated giant cells [20]. It has also been observed that the administration of keratinocyte growth factor (KGF) may increase the viral load by upregulating the expression of ACE2 in these cells [21]. A cytokine storm that includes the release of a high number of various cytokines is also associated with SARS-CoV-2. The accumulation of these inflammatory markers causes vasodilation and increased capillary permeability in the endothelial cells of the smooth muscle, thus causing an influx of fluids into the lungs. The influx of fluids in the interstitial space causes alveolar edema and washout of surfactant produced by type 2 pneumocytes cells, ultimately resulting in an increase in the surface tension of the alveoli and in collapsing pressure. Together, all of these events result in alveolar collapse, decreased gas exchange, and hypoxemia. Additionally, in the presence of pathogenic debris, neutrophils are attracted to the alveoli system where they enhance cell death and ultimately cause hypoxemia [22,23]. The excess release of interleukins ultimately reaches the hypothalamus through the blood system and results in an increase in body temperature (i.e., fever). The body tries to efflux the excess debris containing pathogen and dead cells by transporting it with mucous (i.e., cough). Hypoxemia, fever, and cough are the most important symptoms observed in this disease. There is still a lack of knowledge regarding COVID-19 pathogenesis, and it remains unclear if the entry or propagation of SARS-CoV-2 is the same as that of SARS-CoV or if there are any other known receptors for viral entry. Moreover, the apical cilia present on airway cells and microvilli of type II cells can also facilitate the entry of the virus.

## 3. Treatment of SARS-CoV-2 with Mesenchymal Stem Cells 

Mesenchymal stem cells (MSCs) originate from the bone marrow, umbilical cord, placenta, and other tissues and possess differentiation and programming potential with strong immunomodulation and endogenous repair mechanisms. In regenerative medicine, adult MSCs are known to be protective against pulmonary, neurological, hepatic, renal, and cardiovascular diseases. MSCs are known to exert immunomodulatory effects that impact macrophages and promote polarization towards a therapeutic or reparative phenotype [24]. It was evident from a previously published study that CCL2 (a cytokine responsible for the recruitment of macrophages) derived from MSCs exhibited therapeutic effects by assisting macrophage repolarization [24]. MSCs act as immune modulators for both the innate and the adaptive immune systems. These cells are also involved in the switching of the phenotypes of inflammatory macrophages (M1) towards a reparative or therapeutic phenotype (M2), and they are related to decreased TNF-α and increased IL-10 production that is mediated by cellular contact [24].

Immune modulation of MSCs relies upon the activation of effector functions in immune cells. In a previously published study, MSCs were found to suppress lung infiltration and the dissolution of pulmonary edema. MSCs are multipotent, regenerative, and self-renewable and possess the ability to suppress the immune response and to differentiate into type II alveolar epithelial cells in vitro [25]. In a phase 2 clinical trial (NCT03608592) examining patients with ALI and ARDS, MSCs were demonstrated to possess anti-inflammatory activity [26]. Based on the outcome of this safety trial, MSCs may alleviate SARS-CoV-2-associated CS and ARDS and may also prove to be a prospective therapy for chronic respiratory dysfunction and lung fibrosis. MSCs are the prime therapeutic approach for treatment of lungs fibrosis, because they are easy to isolate, and have minimum immunogenicity, reparative characteristics along with anti-inflammatory properties. Clinical trials proved that MSCs possess short-term safety and tolerability with some limited studies focusing on adverse pro-inflammatory and myofibroblastic feature. The possible mechanism behind the protective effect of MSCs in lung fibrosis is that, MSCs migrate to the site of action (i.e., site of injury) and release multiple paracrine factors that are largely mediated by their extracellular vesicles that regulate the epithelial and endothelial permeability and cause decrease inflammation along with promotion of tissue repair. MSCs exclusively exhibit three characteristics in SARS-CoV-2-infected patients, including (i) activation of T cells to relieve excessive immune responses, (ii) maintenance of homeostasis in response to specific injuries associated with the lungs while also promoting regeneration, and (iii) inhibition of inflammation caused by the release of cytokines that is mediated by extracellular vesicles (EVs) or exosomes [27,28]. These exosomes deliver mRNA, microRNA, DNA, proteins, and metabolites to trigger reparative, therapeutic, and regenerative functions in the lungs [29]. 

## 4. Exosome: A Nanometer-Sized Envelope Possessing Miraculous Properties

Exosomes are nanometer-sized enveloped small extracellular vesicles that are extracted by ultracentrifugation of spent culture media of cells, and they are generated by the processes of the endocytosis pathway [30]. Almost all cell types release exosomes that contain varied biological information in the form of microRNAs (miRNAs), nucleic acids, proteins, growth factors, and EVs. In addition to facilitating cell-to-cell communication, exosomes are also helpful in determining the cell state by acting as biomarkers for diagnosis and prognosis [31]. Exosomes possess a small cup-shaped spherical morphology with sizes ranging from 30 to 100 nm in diameter. Exosomes also contain conserved proteins, including tetraspanins (CD9, CD63, and CD 81), MHC class I and II, and heat shock proteins (HSP 60, 70, and 90) [32]. Primary cells, hematopoietic cells, viral particles, and cancer cells found in biological fluids such as saliva, synovial fluid (SF), serum, plasma, bronchoalveolar lavage (BAL) fluid, urine, amniotic fluid (AF), pleural effusions (PE), semen, were all found in proximity to exosomes.

## 5. Biogenesis of Exosomes

Exosome biogenesis is a continuous cellular phenomenon that is initiated by inward invagination of the plasma membrane within the cytosol to form early and late endosomes. Late endosomes can further fuse together to form multivesicular bodies (MVBs) that invaginate to form intraluminal vesicles (ILVs). ILVs formed during the episodes of inward invagination are accompanied by several essential proteins, cytoskeletal components, nucleic acids, growth factors, necessary cellular components. After formation, these MVBs containing ILVs fuse together with the plasma membrane of the cells and are released as exosomes by exocytosis into the extracellular spaces. In previous studies, it was speculated that in the MVB biogenesis process, the budding of exosome cargos and their sorting is either endosomal sorting complex required for transport (ESCRT)-dependent or -independent [32]. Figure 1 demonstrates the mechanism of EVs biogenesis and its isolation.

It is believed that the ESCRT-mediated cargo-sorting phenomenon includes the identification and sequestration of ubiquitinated proteins to specific sites of the endosomal membranes [32]. Furthermore, the sequential association between subunits I, II, and III of ESCRT terminates the complex and initiates the budding process. It was revealed that the sorting protein Vps4 is involved in the detachment of the ESCRT III complex from the MVBs membrane that aids in the formation of ILVs from cleaved buds [32]. However, ESCRT-independent processes involve proteins and lipids such as tetraspanins (CD81) and ceramides. These exosomes associate post-release with recipient cells for efficient uptake. The exosome uptake mechanism involves both clathrin-dependent and -independent events, micropinocytosis, phagocytosis, and lipid raft-mediated internalization.

These exosomes are composed of proteins such as heat shock proteins, cell adhesion proteins, tetraspanin membrane proteins, cell signaling proteins, transcription proteins, and trafficking membrane fusion proteins [32]. A variety of lipid molecules, including phosphatidylserine (PS), phosphatidic acid, cholesterol, sphingomyelin (SM), arachidonic acid and other fatty acids, prostaglandins, and leukotrienes are also present in these exosomes [32]. In addition to these proteins and lipids constituents, exosomes are also rich in non-coding RNAs such as micro-RNA, small nuclear RNA, small nucleolar RNA, long non-coding RNA, piwi-interacting RNA, rRNA, and tRNA [32]. 

## 6. Mesenchymal Stem Cell-Derived Exosomes 

These exosomes play vital autocrine/paracrine intercellular communication roles via the transfer of biological information to recipient cells. Their nature depends on their origin. For example, exosomes derived from MSCs have been shown to promote more therapeutic/regenerative activities compared to those promoted by MSCs alone. Animal model-based studies examining previously recommended that exosomes allow for the identification of better therapeutic candidates and provide a novel alternative to whole MSC cell-based therapy. These benefits may be attributed to the high safety and long shelf lives associated with exosomes that make them suitable and strong candidates for regenerative medicines and therapeutics for a variety of diseases [33]. It has been observed that MSC-derived exosomes inhibit pro-inflammatory mechanisms and are also associated with the alleviation of oxidative stress and pulmonary fibrosis and the remodeling that occurs in inflammatory lung disease [33]. 

Exosomes function according to their lineages, where their function is dependent upon the phenotype of their parental cell type. It has been reported that MSC-derived exosomes possess the potential to restore and maintain homeostasis based on their capability for protein and RNA transfer [34]. MSC-derived exosomes express several phenotypic markers, including CD29, CD73, CD44, and CD105 [35]. In addition to these phenotypic markers, MSC-derived exosomes also possess surface receptors (PDGFRB, EGFR, and PLAUR), cell adhesion molecules (FN1, EZR, IQGAP1, CD47, integrin, and LGALS1/LGALS3), signaling molecules (RRAS/NRAS, MAPK1, GNA13/GNG12, CDC42, and VAV2), and MSC-associated antigens (CD9, CD63, CD81, CD109, CD151, CD248, and CD276) [36]. It has been reported that MSC-derived exosomes contain more than 850 unique gene products and more than 150 miRNAs [37,38]. 

## 7. Anti-Inflammatory Effect of MSC-Derived Exosomes

It is evident that ARDS is the primary cause of death in individuals with COVID-19. In a previous study, 41 SARS CoV-2 patients were admitted to the hospital during the early outbreak, and 6 of these patients died due to ARDS [39]. ARDS is a common syndrome associated with SARS-CoV-2, SARS-CoV, and MERS-CoV infection and is characterized by a cytokine storm that includes the release of pro-inflammatory marker cytokines such as IFN-α, IFN-γ, IL-1β, IL-6, IL-12, IL-18, IL-33, TNF-α, TGFβ, and chemokines (CCL2, CCL3, CCL5, CXCL8, CXCL9, CXCL10) [39,40,41,42].

MSC-derived exosomes have been identified to be promising for the treatment of ALI, ARDS, inflammatory lung diseases, silicosis, idiopathic pulmonary fibrosis (IPF), chronic obstructive pulmonary disease (COPD), pulmonary artery hypertension, asthma, pneumonia, and bronchopulmonary dysplasia. It is important to elucidate the exact mechanism underlying the therapeutic role of these exosomes in lung diseases. It has been speculated that specific micro-RNAs (miRNAs) are responsible for this effect. Current clinical trials examining anti-inflammatory drugs used in combination with glucocorticoids have demonstrated unsuccessful outcomes in the treatment of lung diseases. MSC-derived exosomes are gaining attention as promising prospective therapies for ALI/ARDS. Zhu administered human bone marrow (HBM) MSC-derived exosomes intratracheally in an ALI mouse model and observed a significant reduction in lung inflammation, pulmonary edema, neutrophil infiltration, macrophage inflammatory protein-2 levels in BALF, and protein permeability [43]. It has also been reported that HBM MSC-derived exosomes can help to relieve inflammatory responses in ARDS by facilitating mitochondrial transfer [44]. It has also been reported that BM MSC-derived exosomes improve the survival outcome of patients with ALI [45].

It has been reported that administration of BM-MSC-derived exosomes in the context of IPF can block TGF-1-induced myofibroblastic differentiation [46]. It has also been observed that MSC-derived exosomes are rich in miRNAs such as miR-34a, 122, 124, and 127 that can facilitate increased levels of anti-inflammatory and anti-proliferative activity [47,48]. In recent years, MSCs have been used to ameliorate immune mediated disorders in pre-clinical and clinical studies. These studies demonstrated the immunosuppressive role of MSCs mediated by suppression of T-cell-mediated immune response, B-cell proliferation, immunomodulation of regulatory T-cell, antigen presentation and pro-inflammatory cytokine release. The various preclinical and clinical trials of MSCs as immunomodulatory features have been demonstrated in Table 1. Anti-inflammatory characteristics are demonstrated in Table 2.

## 8. Pro-Angiogenic Role of MSC-Derived Exosomes

Interestingly, exosomes display pro-angiogenic characteristics and can initiate angiogenesis. It was determined that circulating plasma exosomes in glioma patients can enhance the process of angiogenesis [50]. Another study reported that endothelial cell-derived exosomes also initiate the process of endothelial cell invasion and promote capillary formation due to the presence of miR-146a, and this leads to a decrease in metabolic activity in combination with a downregulation of Erbb4, Notch1, and Irak1 [51]. In a previously published study, it was found that BM-MSC-derived exosomes provide cardioprotection due to paracrine factors such as VEGF, FGF, HGF, and IGF [52]. It was also reported that CD34+ exosomes enhance endothelial cell viability, proliferation, angiogenesis, and tube initiation on Matrigel due to the presence of miR-126 and miR-130 [51]. 

It was found that adipose MSC-derived exosomes significantly promote angiogenesis both in vivo and in vitro due to the presence of miR-125 that inhibits the expression of angiogenic inhibitor delta-like 4 (DLL4) by targeting its 3′ untranslated region (UTR) [53]. MSC-derived exosomes have also been established to be beneficial in the context of ischemic diseases by promoting the initiation of angiogenesis. It is believed that these exosomes deliver the bioactive molecule miR-30b that significantly activates the pro-angiogenic potential of these exosomes upon transplantation into HUVECs cells [54]. It has also been observed that exosomes derived from MSCs are rich in miRNAs such as 125a, 30b, 30c, 424, 150, and let-7f, all of which are regulators of angiogenic activities [55,56,57]. A previous study revealed that exosome miRNAs can be used as modulating agents for VEGF expression and can be used as therapeutics in rheumatoid arthritis [58]. The pro-angiogenic properties of MSC-derived exosomes have been demonstrated in the treatment of myocardial ischemia/reperfusion injury and acute myocardial infarction [59,60]. Table 3 clearly demonstrates the studies published in recent years that established the role of exosomes as pro-angiogenic effectors molecule.

## 9. MSC-Derived Exosomes as a Prospective Treatment for COVID-19 

MSC-derived exosomes can serve as an attractive target for treating COVID-19 and its associated complications such as lung injury and ARDS. These exosomes of MSC lineages possess immunomodulatory, tissue repair, and antiviral properties. A recent study suggested that MSC-derived exosomes can easily be substituted for MSCs, as these exosomes exert the same effects in the treatment of COVID-19 as do MSCs [61]. In another study, it was demonstrated that MSC-derived exosomes could inhibit the influenza virus due to their ability to transfer miRNAs and mRNA into lung epithelial cells to aid in the reduction of cellular apoptosis and viral replication [62]. In a study conducted in 2018, it was observed that MSC-derived exosomes induced a reduction in TNF-α, IL-1β, NF-κB, and matrix metalloprotease 9 (MMP-9) levels in the lung and caused decreased expression of IL-6 gene and increased production of IL-10 [63]. In another study, it was found that the transfer of miR-146a via exosomes exerted immunomodulatory effects through IL-1β augmentation [64].

Researchers are now discovering potential therapies against SARS-CoV-2 infection that include COVID-19 treatment based on MSCs or their derived exosomes [65,66,67,68]. As the pathogenesis of SARS-CoV-2 is similar to that of most of the previously encountered viruses that cause ARDS and lung injury, it has been proposed that based upon previous treatment approaches using MSCs or MSC-derived exosomes, the usefulness of exosome therapy should be explored in the context of SARS-CoV-2.MSC-derived exosomes were previously reported to elicit a positive response in ARDS and to inhibit cytokine storms by transferring mRNA and miRNAs to lung tissues [69,70]. In a preclinical model of non-infectious acute lung injuries or bacterial sepsis in the lungs, MSC-derived exosomes have shown protective effects mediated by miRNAs and inhibitory mRNAs [71]. It was suggested that pulmonary fibrosis of the lungs can be prevented in COVID-19-induced pneumonia through the use of MSC-derived exosomes. The rigorous anti-inflammatory activities of MSC exosomes can be used as an infusion in COVID-19 confirmed cases to prevent inflammatory responses. 

Exosomes have been evaluated previously in the context of SARS-related coronavirus infection, and it was determined that exosomes containing the SARS-CoV spike protein “S” generate neutralizing antibody titers [72]. Kuate injected xenogenic exosomes without any adjuvant into SARS coronavirus-infected subjects and observed the presence of sufficient neutralizing antibodies [72]. In one recent clinical trial (NCT04276987), MSC-derived exosomes were found to be capable of treating severe subjects with novel coronavirus-associated pneumonia when used as an aerosol upon inhalation [73]. In a recently published prospective non-randomized open-label cohort study, the efficacy of allogenic BM-MSC-derived exosomes in the treatment of severe COVID-19 was tested in 24 SARS-CoV-2-infected subjects with moderate-to-severe acute respiratory distress syndrome [74]. A single injection of 15 mL was administered to the enrolled subjects intravenously, and the effects were monitored from days 1 to 14 post exosome injection [74]. A survival rate of 83% was observed, and out of 24 patients, 17 recovered, 3 remained critically ill (although stable), and 4 died due to unrelated treatment [74]. The authors concluded that exosomes derived from MSC lineages can provide a promising candidate for therapy in SARS-CoV-2 infection [74]. In another Chinese clinical trial, it was found that human MSCs and their derived exosomes could aid in the treatment of lung injury associated with novel coronavirus pneumonia (COVID-19) [75]. In a recently active clinical trial (NCT04389385), COVID-19-specific T-cell-derived exosomes were used via a metered-dose inhaler to treat pneumonia associated with SARS-CoV-2 infection [76]. Table 4 shows the prospective role of mesenchymal stem cell-mediated exosomes in SARS-CoV-2 infection [5,77,78,79,80,81,82,83,84,85,86,87,88,89,90,91,92,93,94,95,96,97,98,99,100,101,102,103,104,105,106,107,108,109,110,111,112,113,114]. 

## 10. Management of SARS-CoV-2-Associated Respiratory Dysfunction with Extracellular Vesicles

The most serious complication associated with SARS-CoV-2 infection is respiratory dysfunction that ultimately leads to acute hypoxemic respiratory failure. Previous studies have indicated the presence of substantial hypoxemia in many affected patients [115]. Respiratory dysfunction includes pulmonary edema, vascular occlusion, hemoglobinopathies, and miscommunication between ventilation and perfusion. In one previously published study, it was observed that ARDS is associated with diffuse alveolar damage in combination with microvascular and macrovascular thrombi [116,117]. The heterogeneity of ARDS has been reported previously, where inflammation and positive end-expiratory pressure responses (PEEPR) have been observed. Based on this, it is likely that the pathology of COVID-19 patients is similar to that of ARDS. MSC-derived extracellular vesicles (EVs) are known to play anti-inflammatory and immunosuppressive roles in the treatment and management of inflammation and the repair of damaged tissues. The beneficial approach of MSC-derived EVs involves the immune modulation of cells, the anti-bacterial properties of these EVs, and capillary barrier function restoration [118]. In previous studies, it was reported that MSCs elicited beneficial effects by interacting with the lung microenvironment in a manner that is mediated through paracrine signaling. To accomplish this, MSCs release EVs, which inhibit the release of TNF-α and IL-6, increase the secretion of IL-10, keratinocyte growth factor (KGF), and angiopoietin-1, facilitate macrophage reprogramming functions, and release antimicrobial peptides [119]. Shah et al. demonstrated that endogenous BM-MSC-derived exosomes express Runx-1 and TGF-β receptor-1 transcription factors that influence the outcome of patients with ARDS [120]. These remarkable beneficial properties of MSC-derived exosomes can be used as a prospective therapy for the management and treatment of SARS-CoV-2-associated lung damage. Table 5 and Table 6 show the anti-fibrotic and immunomodulatory role of exosomes that can be proving beneficial in the treatment and management of the COVID-19.

## 11. Delivery Route for Efficient Treatment against SARS-CoV-2

MSC-derived exosomes still require further clarification in regard to determining an effective delivery route to SARS-CoV-2 positive subjects as a treatment strategy. Effective use of exosomes for the treatment of inflammatory lung diseases has been achieved through intravenous and intratracheal injections. In one previously published study, bioluminescence and fluorescence-mediated tomography imaging results indicated that exosomes injected intravenously were localized to the liver, lungs, spleen, and kidneys, with some traces in the brain, heart, and muscles within 30 min of injection [121]. It has been reported that intratracheal delivery of exosomes or biological therapeutics in clinical use has some advantages over systemic delivery [122].

In recent clinical trials of MSCs and their derivatives for the treatment of SARS-CoV-2, intravenous injection was preferred. Moreover, it was understood that intravenous delivery does not guarantee a specific lung target approach, and therefore, inhalation provides significant relief to SARS-CoV-2-infected subjects, as inhalation of these exosomes through aerosol may result in direct and specific delivery to the lungs to provide a more effective means of treating lung-associated complications. Although inhalation appears to be a facile method by which to target the lungs, it is also challenging in terms of uniform distribution in cells. In a recent clinical trial, exosomes or extracellular vesicles were administered through the inhalation route to patients with severe pneumonia associated with SARS-CoV-2 infection (NCT04276987) [73]. The authors reported that the inhalation delivery mode of the exosomes prevents aggregation of these biological molecules within the injured microcirculation compared to that observed in response to the intravenous delivery mode [73]. In another recently published study examining pulmonary fibrosis, the inhaled delivery mode of spheroid cell-secretomes and exosomes for lung regeneration exhibited promising results [123]. Importantly, regardless of the delivery routes and types of stem cells, new clinical trials are required that incorporate a placebo arm and patient randomization to achieve maximum efficacy of exosomes in the treatment of COVID-19.

## 12. Non-EVs and Non-Vaccine-Based Treatment Regime in SARS-CoV-2

SARS-CoV-2 treatment regimens includes uses of antivirals, systemic steroids, monoclonal antibodies, anti-inflammatory agents, antibiotics, and convalescent plasma. Hydroxychloroquine/Chloroquine is among the favorite and economically cheaper candidate involved in reducing the viral nucleic acid production and multiplication by inhibiting the binding of spike (S) protein to the ACE2 receptors that further limit the cytokine storm [124]. Similarly, other drugs including Remdesivir, Lopinavir/ritonavir and Favipiravir also showed promising approach in the management of the COVID-19-associated co-morbidities [124]. Monoclonal antibodies like Tocilizumab, Sarilumab, and Bevacizumab showed a beneficial approach in reduction of severity of the COVID-19 disease by restricting the IL-6 production and limiting cytokine storm [124]. Another approach is based on the use of immune enhancers like intravenous gamma globulins, NK cells, interferons have also showed promising results in treatment of COVID-19 severity [124]. Moreover, use of systemic steroids have also showed a beneficial role in lessening the disease severity [124].

## 13. Conclusions

The term “exosome” refers to cell-derived vesicles that possess diversity in regard to their biogenesis and biological properties. The role of BM-MSC-derived exosomes in the pathogenesis of diseases has been extensively studied in the context of various diseases. COVID-19 is a global pandemic caused by SARS-CoV-2 infection that is characterized by lung-associated complications, and the entry of this virus is mediated by ACE-2 receptors. MSC-derived exosomes possess the therapeutic potential to treat SARS-CoV-2-associated lung injury, particularly pneumonia, due to their immunomodulatory and pro-angiogenesis roles. These exosomes are rich in several miRNAs that regulate the expression of a number of transcription factors responsible for lung damage and protection. The outcome of SARS CoV-2-infected patients can be improved using exosomes, as previously demonstrated in the context of ARDS. Pre-clinical and clinical trials have shown that the prospective role of MSCs is mediated by the paracrine effect of exosomes in COVID-19. Further preclinical and clinical studies are required to fully clarify the specific mode of delivery of these exosomes in an effort to mitigate COVID-19-associated risk factors. The focus should be more on production of the clinical grade exosomes that can be further exploited for the purpose of disease interventions. Bioreactor-based large-scale clinical grade exosomes approaches can be employed under the good manufacturing practice (GMP) standard guidelines followed by in vitro and in vivo clinical testing of the generated exosomes or extracellular vesicles in treatment of the disease. Furthermore, these GMP-produced exosomes should be checked for their shelf life, bio-distribution and efficiency in treatment of COVID-19 and its associated co-morbidities.

Take Home Message:

BD-MSC-derived exosomes should be explored for the treatment of SARS CoV-2-associated lung damage.

Exosomes from SARS-CoV-2-infected subjects can also be used as prospective biomarkers for assessing the degree of lung damage.

Exosomes derived from sources other than BM-MSCs should be explored further.

## Figures and Tables

**Table 1:** List of clinical trials of mesenchymal stem cells (MSCs)-based therapy of autoimmune disorders.

Disease	Source of MSCs	Phase	First Posted Date, Initiating Company/Sponsor, Country
Chronic Autoimmune Urticaria	Auto AD-MSC	Phase 1	03.03.2017; Celal Bayar University, Turkey.
Multiple Sclerosis	Auto AD-MSC	Phase 1 Phase 2	01.2010; Regional University Hospital in Málaga, University Hospital Virgen Macarena, Spain.
			30.12.2014; American CryoStem Corporation, Cayman Islands.
	AD-MSC, no specification	Phase 1 Phase 2	04.10.2018; Stem Cell Medicine Ltd., Israel.
	Auto MSC	Phase 1 Phase 2	21.11.2012; Karolinska Institute, Stockholm, Sweden.
			16.05.2013; University of Genova, Italy.
			12.09.2014; Ottawa Hospital Research Institute, Canada.
			26.10.2010; Instituto de Salud Carlos III, Spain.
			23.12.2008; Cleveland Clinic Mellen Center, USA.
			25.05.2012; Imperial College London, United Kingdom.
	UC-MSC	Phase 1 Phase 2	25.07.2017; Jordan University Hospital Amman, Jordan.
			27.10.2015; Novo Cellular Medicine Institute LLP, India.
			16.04.2015; Genesis Limited, India.
	Auto BM-MSC	Phase 1 Phase 2	31.03.2015; University Hospital, Toulouse, France.
			18.12.2018; Karolinska Institute, Sweden.
			21.06.2011; Royan Institute, Iran.
			14.01.2014; Germans Trias i Pujol Hospital Badalona, Spain.
			13.07.2015; Vall d’Hebron Research Institute, Spain.
			10.12.2012; Andalusian Initiative for Advanced Therapies, Spain.
			29.10.2008; Hadassah Medical Organization, Israel.
			18.06.2014; Hadassah Medical Center, Israel.
			03.03.2017; Stem Cells Arabia, Jordan.
			10.07.2013; Jordan University Hospital Amman, Jordan.
			10.01.2019; Brainstorm-Cell Therapeutics, USA.
			02.11.2006; University of Cambridge, United Kingdom.
Crohn’s Disease	Allo BM-MSC	Phase 1 Phase 2	22.02.2012; University Hospital Liège, Belgium.
	UC-MSC	Phase 1 Phase 2	04.12.2013; Kang Stem Biotech Co., Ltd., South Korea.
Rheumatoid Arthritis	Auto AD-MSC	Phase 1 Phase 2	02.10.2018; Hope Biosciences, USA.
	Allo AD-MSC	Phase 1 Phase 2	13.08.2012; TiGenix S.A.U., Spain.
	Auto BM-MSC	Phase 2 Phase 3	10.06.2013; Royan Institute, Iran.
		Phase 1	20.06.2016; Mashhad University of Medical Sciences, Iran.
			14.06.2017; MetroHealth Medical Center, USA.
			01.03.2017; Stem Cells Arabia, Jordan.
	UC-MSC	Phase 1 Phase 2	13.01.2014; Stem Cell Institute, Panama.
			15.11.2013; Translational Biosciences, Panama.
			07.03.2012; Alliancells Bioscience Corporation Limited, China.
			31.12.2015; Shenzhen Hornetcorn Bio-technology Company, LTD, China.
			04.02.2019; Baylx Inc., USA.
			07.08.2018; Kang Stem Biotech Co., Ltd., South Korea.
Systemic Lupus Erythematosus	UC-MSC in Plasma-Lyte A solution	Phase 2	17.12.2015; Medical University of South Carolina, USA.
	MSCs from UC, BM, AD, and DP and other in Plasma-Lyte A solution.	Phase 1	31.05.2017; Medical University of South Carolina, USA.
	Allo BM-MSC	Phase 1 Phase 2	17.06.2008; Nanjing Medical University, China.
	UC-MSC	Phase 1 Phase 2	05.12.2012; Nanjing Medical University, China.
			01.07.2018; Saint-Louis Hospital, France.
Lupus Nephritis	Allo BM-MSC	Phase 1 Phase 2	17.09.2018; University Hospital Río Hortega, Spain.
			02.06.2017; Corestem, Inc., South Korea.
	Auto MSCs	Phase 1 Phase 2	16.04.2008; Organ Transplant Institute, China.
	UC-MSCs	Phase 2	09.07.2018; Lingyun Sun, China.
			28.02.2012; CytoMed & Beike, China.
Type 1 Diabetes	Auto BM-MSCs	Phase 1 Phase 2	25.03.2011; University of Sao Paulo, Brazil.
			05.12.2017; Central Hospital, Nancy, France.
	Auto BM-MSC	Phase 2 Phase 3	07.07.2010; Southwest Hospital, Third Military Medical University, China.
	UC-MSCs	Phase 1 Phase 2	13.10.2010; Stem Cell Research Center of Medical School Hospital of Qingdao University, China.
			16.06.2011; Fuzhou General Hospital, China.
			2.06.2011; Shenzhen Beike Bio-Technology Co., Ltd., China.
			05.05.2016; The Affiliated Nanjing Drum Tower Hospital of Nanjing University Medical School, China.
	Cotransplantation of allograft Islet and auto MSCs	Phase 1 Phase 2	28.03.2008; Fuzhou General Hospital, China.
	Auto MSCs	Phase 2	07.02.2014; Uppsala University Hospital, Sweden.
	Auto BM-MSC and Allo UC-MSC combined with PRP	Phase 1 Phase 2	01.04.2017; Van Hanh General Hospital University of Science, Vietnam.
	Allo BM-MSC	Phase 2	08.09.2016; Clínica Alemana de Santiago, Chile.
	Allo AD-MSC with auto BM-MSC	Phase 1	20.10.2016; Sophia Al-Adwan, University of Jordan, Jordan.
	Auto BM-MSC and UC-MSCs	Phase 1	14.06.2010; Cellonis Biotechnology Co. Ltd., China.
	PROCHYMAL® - adult MSCs	Phase 2	04.06.2008; Mesoblast International, USA.
	Men-MSCs	Phase 1 Phase 2	21.12.2011; Evans Biosciences Co., Ltd., Zhejiang University, China.
Neuromyelitis Optica Spectrum Disorders	Auto MSCs	Phase 2	25.09.2014; Tianjin Medical University General Hospital, China.
Sjögren’s Syndrome	Allo MSCs	Phase 1 Phase 2	06.08.2009; The Affiliated Nanjing Drum Tower Hospital of Nanjing University Medical School, China.
Hepatitis	UC-MSCs	Phase 1 Phase 2	10.08.2012; Beijing 302 Hospital, China.

UC—umbilical cord; BM—bone marrow; AD—adipose-derived; DP—dental pulp; auto AD-MSCs—autologous adipose-derived mesenchymal stem cells; Allo AD-MSC—allogenous adipose-derived mesenchymal stem cells; auto BM-MSCs—autologous bone-marrow-derived mesenchymal stem cells; Allo BM-MSCs—allogenous bone-marrow-derived mesenchymal stem cells; UC-MSCs—umbilical cord-derived mesenchymal stem cells; Allo UC-MSC—allogenous umbilical cord-derived mesenchymal stem cells; PRP—platelet-rich plasma. (Adopt under the terms of the Creative Commons Attribution License (CC BY) from Gomzikova Marina O., James Victoria, Rizvanov Albert A. Therapeutic Application of Mesenchymal Stem Cells-Derived Extracellular Vesicles for Immunomodulation. Frontiers in Immunology, 10,2019, 2663.10.3389/fimmu.2019.02663) [49].

**Table 2:** Studies showing mesenchymal stem cells as therapy in COVID-19.

Articles	Conclusion of the Study	References
Mesenchymal stem cells as a potential therapy for COVID-19	A summary of clinical trials of MSCs treatments on ALI/ARDS and raise MSCs as a hopefully alternative therapy for COVID-19.	[1]
Mesenchymal Stem Cell Therapy for COVID-19: Present or Future	Study of MSCs-based improvement in patient’s immunological responses to COVID-19.	[2]
Combating COVID-19 with mesenchymal stem cell therapy	Highlights implications associated with MSC therapy application in case of COVID-19	[3]
Mesenchymal stem cells and management of COVID-19 pneumonia	Collection of studies suggesting improving patient’s biological resistance to COVID-19 using stem cells	[4]
Transplantation of ACE2 - Mesenchymal Stem Cells Improves the Outcome of Patients With COVID-19 Pneumonia	The intravenous transplantation of MSCs was reliable, safe and efficient for treatment of COVID-19 pneumonia, especially for the patients in critically severe condition.	[5]
Clinical remission of a critically ill COVID-19 patient treated by human umbilical cord mesenchymal stem cells (hUC-MSCs)	The adoptive transfer therapy of hUC-MSCs might be an ideal choice to be used or combined with other immune modulating agents to treat the critically ill COVID19 patients.	[6]

**Table 3:** Studies showing the anti-inflammatory role of mesenchymal stem cells-derived exosomes.

Articles	Conclusion of the Study	Reference
Mesenchymal stem cells secrete immunologically active exosomes	Infusion of MSC exosomes enhanced the survival of allogenic skin graft in mice and increased Tregs.	[1]
Mesenchymal stem cells-derived exosomes are more immunosuppressive than microparticles in inflammatory arthritis	Demonstration of the therapeutic potential of MSCs-derived EVs in inflammatory arthritis.	[7]
Exosomes in mesenchymal stem cells, a new therapeutic strategy for cardiovascular diseases?	Recent advances about the role of exosomes in MSCs therapy for CVDs.	[8]
Mesenchymal stem cells-derived exosomes (Exos) and microparticles (MPs) protect cartilage and bone from degradation in osteoarthritis	MPs and Exos used related chondroprotective and anti-inflammatory function in vitro and protected mice from developing OA in vivo, suggesting that either Exos or MPs reproduced the main therapeutic effect of BM-MSCs.	[9]
Mesenchymal stem cell-derived exosomes improve the microenvironment of infarcted myocardium contributing to angiogenesis and anti-inflammation	Exosomes stimulate neovascularization and restrain the inflammation response, thus improving heart function after ischemic injury.	[10]
Exosomes derived from mesenchymal stem cells	This paper provides a general overview of MSC-exosomes as a new cell-free therapeutic paradigm.	[11]
Mesenchymal stem cells in regenerative medicine applied to rheumatic diseases: role of secretome and exosomes	Strategies for the development of the MSC secretome with respect to the release of extracellular vesicles that would have certain advantages over injection of living MSCs or administration of a single therapeutic factor or a combination of factors.	[12]
Pro inflammatory stimuli enhance the immunosuppressive functions of adipose mesenchymal stem cells-derived exosomes	Study suggests that the immunomodulatory properties of AMSCs-derived exosomes may be not constitutive, instead induced by the inflammatory microenvironment.	[13]
Immunomodulatory effects of mesenchymal stromal cells-derived exosome	IDO showed no significant changes in PBMCs exposed to MSCs-derived exosome. They conclude that exosomes and MSCs might differ in their immune-modulating activities and mechanisms.	[14]

**Table 4:** Studies showing pro-angiogenic role of mesenchymal stem cells-derived exosomes.

Articles	Conclusion of the study	Reference
Cancer exosomes trigger mesenchymal stem cell differentiation into pro-angiogenic and pro-invasive myofibroblasts	Prostate cancer exosomes dominantly dictate a programme of MSC differentiation generating myofibroblasts with functional properties consistent with disease promotion.	[16]
Mesenchymal stem cells release exosomes that transfer miRNAs to endothelial cells and promote angiogenesis	Exosomal transfer of pro-angiogenic miRNAs plays an important role in MSC-mediated angiogenesis and stem cell-to-endothelial cell communication.	[17]
Exosomes secreted by mesenchymal stem cells promote endothelial cell angiogenesis by transferring miR-125a	adMSC-Exo can transfer miR-125a to endothelial cells and promote angiogenesis by repressing DLL4.	[18]
Exosomes derived from mesenchymal stem cells suppress angiogenesis by down-regulating VEGF expression in breast cancer cells	MSC-derived exosomes may serve as a significant mediator of cell-to-cell communication within the tumor microenvironment and suppress angiogenesis by transferring anti-angiogenic molecules.	[19]
Exosome and mesenchymal stem cell cross-talk in the tumor microenvironment	MSCs have a potential to exert anti-tumor activities, they largely provide service to the tumor using the multidirectional communication system established by exosomes in the TME.	[20]
Exosomes from cardiomyocyte progenitor cells and mesenchymal stem cells stimulate angiogenesis via EMMPRIN	CMPC and MSC exosomes have powerful pro-angiogenic effects, and this effect is largely mediated via the presence of EMMPRIN on exosomes.	[21]
Exosomes secreted by hypoxic cardiosphere-derived cells enhance tube formation and increase pro-angiogenic miRNA	Benefits of hypoxic CDC exosomes for the treatment of cardiac diseases by induction of angiogenesis via enrichment of pro-angiogenic exosomal miRNAs.	[22]
Mesenchymal stem cell exosomes induce proliferation and migration of normal and chronic wound fibroblasts, and enhance angiogenesis in vitro	MSC exosomes were found to activate several signaling pathways important in wound healing (AKT, ERK, and STAT3) and induce the expression of a number of growth factors [hepatocyte growth factor (HGF), insulin-like growth factor-1 (IGF1), nerve growth factor (NGF), and stromal-derived growth factor-1 (SDF1)].	[23]
Exosomes secreted by human-induced pluripotent stem cell-derived mesenchymal stem cells attenuate limb ischemia by promoting angiogenesis in mice	Implanted iMSCs-Exo was able to protect limbs from ischemic injury via the promotion of angiogenesis, which indicated that iMSCs-Exo may be a novel therapeutic approach in the treatment of ischemic diseases.	[24]
Exosomes from hypoxia-treated human adipose-derived mesenchymal stem cells enhance angiogenesis through VEGF/VEGF-R	Exosomes from hypoxia-treated human ADSCs possess a higher capacity to enhance angiogenesis in fat grafting, at least partially, via regulating VEGF/VEGF-R signaling.	[25]

**Table 5:** Studies showing anti-fibrotic role of mesenchymal stem cells-derived exosomes.

Articles	Conclusion of the Study	Reference
Mesenchymal stem cells deliver exogenous microRNA-let7c via exosomes to attenuate renal fibrosis	The effective antifibrotic function of engineered MSCs is able to selectively transfer miR-let7c to damaged kidney cells and will pave the way for the use of MSCs for therapeutic delivery of miRNA targeted at kidney disease.	[26]
Exosomes derived from miR-181-5p-modified adipose-derived mesenchymal stem cells prevent liver fibrosis via autophagy activation	The effective anti-fibrotic function of engineered ADSCs is able to selectively transfer miR-181-5p to damaged liver cells and will pave the way for the use of exosome-ADSCs for therapeutic delivery of miRNA targeting liver disease.	[27]
Mesenchymal stem cells in regenerative medicine applied to rheumatic diseases: role of secretome and exosomes	Strategies for the development of the MSC secretome with respect to the release of extracellular vesicles that would have certain advantages over injection of living MSCs or administration of a single therapeutic factor or a combination of factors.	[12]
Exosomes derived from human umbilical cord mesenchymal stem cells alleviate liver fibrosis	The hUC-MSC-Exo could ameliorate CCl4-induced liver fibrosis by constraining EMT and protecting hepatocytes	[103]
Mesenchymal stem cells-derived exosomes and microparticles protect cartilage and bone from degradation in osteoarthritis	MPs and Exos used related chondroprotective and anti-inflammatory function in vitro and protected mice from using OA in vivo, suggesting that either Exos or MPs reproduced the main therapeutic effect of BM-MSCs.	[9]
Fibroblasts rendered antifibrotic, antiapoptotic, and angiogenic by priming with cardiosphere-derived extracellular membrane vesicles (CSp-EMVs)	CSp-EMVs alter fibroblast phenotype and secretome in a salutary positive-feedback loop. The phenotypic conversion of inert cells to therapeutically active cells uncovers a novel mechanism for amplification of exosome bioactivity.	[28]
Antifibrotic, antioxidant, and immunomodulatory effects of mesenchymal stem cells in HOCl-induced systemic sclerosis (SSc)	This work shows the beneficial and systemic effects of MSC administration in the HOCl murine model of diffuse SSc, which is a promising finding from a clinical perspective.	[29]
Human bone marrow mesenchymal stem cells-derived exosomes (hBM-MSC-Exo) alleviate liver fibrosis through the Wnt/β-catenin pathway	The hBM-MSCs-Exo treatment could ameliorate CCl4-induced liver fibrosis via inhibition of HSC activation through the Wnt/β-catenin pathway.	[30]

**Table 6:** Studies showing immunomodulatory role of mesenchymal stem cells-derived exosomes.

Articles	Conclusion of the Study	Reference
Immunomodulatory potential of human adipose mesenchymal stem cells-derived exosomes on in vitro stimulated T cells	MSCs-derived exosomes are a cell-derived product that could be considered as a therapeutic agent for the treatment of inflammation-related diseases.	[31]
Immunomodulatory effects of mesenchymal stromal cells-derived exosome	IDO showed no significant changes in PBMCs exposed to MSCs-derived exosome. They conclude that exosome and MSCs might differ in their immune-modulating activities and mechanisms.	[14]
Mesenchymal stromal cell exosomes ameliorate experimental bronchopulmonary dysplasia and restore lung function through macrophage immunomodulation	The MSC-derived exosome mechanism of action is associated with modulation of lung macrophage phenotype.	[32]
Mesenchymal stem cell and derived exosome as small RNA carrier and Immunomodulator to improve islet transplantation	This work elucidated the mechanisms of RNA delivery from hBMSCs to human islets and the immunosuppressive effect of hBMSC and peripheral blood mononuclear cell co-cultured exosomes for improving islet transplantation.	[33]
Mesenchymal stem cell-derived exosomes: immunomodulatory evaluation in an antigen-induced synovitis porcine model	The study suggested immunomodulatory effect of the exosomes and pointed out that they may represent a promising therapeutic option for the treatment of synovitis.	[34]
Immunomodulatory effects of mesenchymal stem cell–derived exosomes on experimental type-1 autoimmune diabetes	AD-MSC’s exosomes exert ameliorative effects on autoimmune T1DM through increasing regulatory T-cell population and their products without a change in the proliferation index of lymphocytes, which makes them more effective and practical candidates.	[35]
Study of immunomodulatory function of exosomes derived from human umbilical cord mesenchymal stem cells (hUC-MSCs)	The hUC-MSCs-exosome has the immunomodulatory function in vitro, which could be a new therapeutic agent for the treatment of immune disorders.	[36]
Primed mesenchymal stem cells package exosomes with metabolites associated with immunomodulation	MSCs exposed to priming culture conditions undergo glycolytic reprogramming, which homogenizes MSCs’ metabolomic profile	[37]
The secretome of mesenchymal stromal cells: role of extracellular vesicles in immunomodulation	Mesenchymal stromal cells influence the cells of the immune system. This influence is mainly due to the release of paracrine factors	[38]
Mesenchymal stem cells secrete immunologically active exosomes	Infusion of MSC exosomes enhanced the survival of allogenic skin graft in mice and increased Tregs.	[1]
Mesenchymal stem cells-derived exosomes (Exos) are more immunosuppressive than microparticles (MPs) in inflammatory arthritis	MSCs-derived MPs and Exos exerted an anti-inflammatory role on T and B lymphocytes independently of MSCs priming.	[88]

## Data Availability

Not Applicable.
